# Comprehensive analysis of key genes associated with ceRNA networks in nasopharyngeal carcinoma based on bioinformatics analysis

**DOI:** 10.1186/s12935-020-01507-1

**Published:** 2020-08-26

**Authors:** Yuanji Xu, Xinyi Huang, Wangzhong Ye, Yangfan Zhang, Changkun Li, Penggang Bai, Zhizhong Lin, Chuanben Chen

**Affiliations:** 1 Department of Radiation Oncology, Fujian Medical University Cancer Hospital & Fujian Cancer Hospital, No. 420, Fuma Road, Fuzhou, 350014 Fujian People’s Republic of China; 2 Fujian Medical University, Fuzhou, Fujian People’s Republic of China; 3 Fujian Normal University, Fuzhou, Fujian People’s Republic of China

**Keywords:** Nasopharyngeal carcinoma (NPC), Bioinformatics analysis, Gene Expression Omnibus (GEO), Differentially expressed genes (DEGs), Gene ontology (GO), Competing endogenous RNA (ceRNA) network

## Abstract

**Background:**

Nasopharyngeal carcinoma (NPC) is an epithelial malignancy with high morbidity rates in the east and southeast Asia. The molecular mechanisms of NPC remain largely unknown. We explored the pathogenesis, potential biomarkers, and prognostic indicators of NPC.

**Methods:**

We analyzed mRNAs, long non-coding RNAs (lncRNAs), and microRNAs (miRNAs) in the whole transcriptome sequencing dataset of our hospital (five normal tissues vs. five NPC tissues) and six microarray datasets (62 normal tissues vs. 334 NPC tissues) downloaded from the Gene Expression Omnibus (GSE12452, GSE13597, GSE95166, GSE126683, and GSE70970, GSE43039). Differential expression analyses, gene ontology (GO) enrichment, kyoto encyclopedia of genes and genomes (KEGG) analysis, and gene set enrichment analysis (GSEA) were conducted. The lncRNA-miRNA-mRNA competing endogenous RNA (ceRNA) networks were constructed using the miRanda and TargetScan database, and a protein–protein interaction (PPI) network of differentially expressed genes (DEGs) was built using Search Tool for the Retrieval of Interacting Genes (STRING) software. Hub genes were identified using Molecular Complex Detection (MCODE), NetworkAnalyzer, and CytoHubba.

**Results:**

We identified 61 mRNAs, 14miRNAs, and 10 lncRNAs as shared DEGs related to NPC in seven datasets. Changes in NPC were enriched in the chromosomal region, sister chromatid segregation, and nuclear chromosome segregation. GSEA indicated that the mitogen-activated protein kinase (MAPK) pathway, phosphatidylinositol-3 OH kinase/protein kinase B (PI3K-Akt) pathway, apoptotic pathway, and tumor necrosis factor (TNF) were involved in the initiation and development of NPC. Finally, 20 hub genes were screened out via the PPI network.

**Conclusions:**

Several DEGs and their biological processes, pathways, and interrelations were found in our current study by bioinformatics analyses. Our findings may offer insights into the biological mechanisms underlying NPC and identify potential therapeutic targets for NPC.

## Background

Nasopharyngeal carcinoma (NPC) is an epithelial malignancy originating from the inner mucosal lining of the nasopharynx [1]. In 2018, there were an estimated 129,079 new cases of NPC worldwide, accounting for 0.7% of all cancer sites, and 72,987 deaths due to NPC, accounting for 0.8% of all cancer sites [2]. The incidence of NPC is geographically imbalanced, with new cases mainly concentrated in east and southeast Asia, especially in South China [3, 4]. The estimated age-standardized incidence rate of NPC is 3.0 per 100,000 in China and only 0.47 per 100,000 in North America [1, 5]. The oncogenesis and progression of NPC are strongly associated with hereditary susceptibility, environmental or random aspects, and Epstein-Barr virus (EBV) infection [6]. In the early stages of NPC, the main pathogenesis is related to EBV infection [7]. Indeed, the expression of EBV-DNA can be used for the monitoring and follow-up of NPC patients, and maybe a useful indicator for risk-stratification strategies [8]. However, despite the great advances in medical technology in recent years, such as the application of intensity-modulated radiotherapy and optimized chemotherapy strategies, the detection and treatment of NPC remain challenging [9]. Epidemiological investigations have shown that although the incidence and mortality rates of NPC have been greatly reduced over the past decade even in endemic areas [10], the survival rate of NPC patients remains unsatisfactory due to local recurrence and distant metastasis, especially in patients with advanced-stage disease [11]. Thus, non-invasive, cancer-specific biomarkers for early diagnosis and precision treatment are urgently required.

Microarray and bioinformatics analyses have enabled researchers to screen the genetic alterations in NPC, and have proven to be convenient methods for identifying potential biomarkers in other diseases. These analytic methods have uncovered several biomarkers with proven prognostic value and potential clinical applications in NPC. For example, one study discovered a novel long non-coding RNA (lncRNA) named LINC01385 involved in NPC development, and functional analysis demonstrated that LINC01385 could serve as a therapeutic target in NPC [12]. Microarray and RNA-sequencing techniques have been used to identify differentially expressed genes (DEGs) and signaling pathways related to the oncogenesis and development of NPC. One study analyzed two microarray datasets to identify DEGs in 14 normal tissue samples and 43 NPC tissue samples; however, the false-positive rate for the two datasets was potentially high, and the limited sample size may have led to unreliable results due to the substantial heterogeneity among the patients [13]. Zhang et al. investigated three microarray datasets to identify DEGs and hub genes that may serve as potential diagnostic biomarkers for NPC [14]. Both these studies analyzed only chip datasets and did not include sequencing data, which would lead to offsets in the studies. Thus, the precise molecular mechanisms and biological processes underlying NPC remain largely unknown, and must be urgently investigated to develop a precise curative treatment for NPC.

In recent years, competitive endogenous RNA (ceRNA) has provided a new way to study the molecular mechanism of cancer. He et al. found that circGFRA1 may serve as ceRNA to regulate GFRA1 expression by sponging mi-34a in triple negative breast cancer [15]. CeRNA is a transcript that can compete shared miRNAs and regulate one another at the post-transcription level, and the ceRNA networks link the function of mRNAs to the function of microRNAs (miRNAs), lncRNAs, circular RNAs and other RNAs [16]. CeRNA can act as miRNA sponges, thereby affecting miRNA expression [17]. CeRNA regulation network refers to the regulatory network with ceRNA participation.

Therefore, in this study, we aimed to explore the molecular pathogenesis, potential biomarkers, and prognostic indicators of NPC by analyzing the full transcriptome sequencing data from Fujian Cancer Hospital along with six microarray datasets acquired from the Gene Expression Omnibus (GEO) to identify DEGs between NPC samples and normal tissue samples. Our findings may guide the precision treatment of NPC.

## Materials and methods

### Sample collection and preparation

Fresh nasopharyngeal tissues were collected from five NPC patients who were treated in Fujian Cancer Hospital between November 2018 and May 2019. Normal nasopharyngeal tissues from five healthy donors were also collected. All tissue samples were frozen using liquid nitrogen. The five NPC patients consisted of three men and two women, with a median age of 41 years. The age and sex of five donors were matched to the NPC patients. Two patients had stage III NPC, while three patients had stage IVA NPC, according to the 8th edition of the current International Union Against Cancer/American Joint Committee on Cancer guidelines for NPC [18]. The ethics committee of Fujian Cancer Hospital approved the human tissue samples related to this work (project ethics number: SQ2019-018-01). The fresh tissue samples were removed from liquid nitrogen and subjected to total RNA extraction using the Trizol method. The purity of the extracted RNA was determined using a Nano Photometer^®^ spectrophotometer (Implen, CA, USA). The RNA concentration was measured using the Qubit^®^ RNA Assay Kit and a Qubit^®^ 2.0 Fluorometer (Life Technologies, CA, USA), and the RNA integrity was evaluated using the RNA Nano 6000 Assay Kit of the Bioanalyzer 2100 system (Agilent Technologies, CA, USA).

### RNA sequencing

A total of 3 μg RNA from each tissue sample served as the input material for the RNA sample arrangements. Ribosomal RNA was eliminated using Epicentre Ribo-zero™ rRNA Removal Kit (Epicentre, USA). The rRNA-free residue was removed using ethanol precipitation. Next, sequencing libraries were produced from the rRNA-depleted RNA by using the NEBNext^®^ Ultra™ Directional RNA Library Prep Kit for Illumina^®^ (NEB, USA). In brief, fragmentations were implemented by bivalent cations below the high temperature in NEBNext First Strand Synthesis Reaction Buffer(5X). The first-strand cDNA thus obtained was compounded using a stochastic hexamer primer and M-MuLV Reverse Transcriptase (RNase H-). Then, second-strand cDNA synthesis was carried out using DNA Polymerase I and RNase H-. In the reaction buffer, dNTPs with dUTP were substituted for dTTP. Residual overhangs were turned into blunt ends through exonuclease/polymerase activities. After the adenylation of the 3′ ends of the DNA fragments, the NEBNext adapter carrying a hairpin loop structure was ligated to initiate hybridization. To optimize the cDNA fragments with a length of 150–200 bp, we purified the library fragments with the AMPure XP system (Beckman Coulter, Beverly, USA). Next, 3 μl USER Enzyme (NEB, USA) was applied with the size-selected, adaptor-ligated cDNA at 37 °C for 15 min and then at 95 °C for 5 min. Thereafter, PCR was conducted using Phusion High-Fidelity DNA polymerase, general PCR primers, and Index (X) Primer. Finally, the obtained products were refined (AMPure XP system), and the library quality was evaluated on the Agilent Bioanalyzer 2100 system. After the library was constructed, Qubit2.0 was used for preliminary quantification; the library was diluted to 1 ng/μl, then used the Agilent 2100 system was used to determine the insert size of the library. After the insert size was confirmed to be as expected, q-PCR was used to confirm the valid concentration (> 2 nM) and accurate quantification of the library to ensure library quality. When the library was deemed eligible, varying libraries were pooled to meet the demands of valid concentration and enable offline data volume. HiSeq sequencing was conducted. These whole-transcriptome sequencing data were termed the FJCH dataset.

### Microarray data

GEO [19] (http://www.ncbi.nlm.nih.gov/geo) is a public genomics dataset repository, which collects high-throughput sequencing data, chips, and microarrays. We downloaded the following six gene expression datasets from GEO: the mRNA gene expression datasets GSE12452 and GSE13597, the miRNA gene expression datasets GSE43039 and GSE70970 and the lncRNA gene expression datasets GSE95166 and GSE126683. All six datasets were annotated using R software (version 3.6.1) via annotation documents. All datasets were from the species Homo sapiens, and the dataset type was microarray expression profile. Details of every dataset study are provided in Table 1.

**Table 1:** Associated microarray datasets from the gene expression omnibus (GEO) database

Reference	PMID	Record	Tissue	Platform	Normal	Cancer
FJCH	/	/	NPC	Illumina HiSeqTM2500/Miseq^TM^	5	5
Dodd et al. [20]	17119049	GSE12452	NPC	[HG-U133_Plus_2] Affymetrix Human Genome U133 Plus 2.0 Array	10	31
Bose et al. [21]	19142888	GSE13597	NPC	[HG-U133A] Affymetrix Human Genome U133A Array	3	25
Zheng et al. [22]	31331909	GSE126683	NPC	Agilent-045997 Arraystar human lncRNA microarray V3 (Probe Name Version)	3	3
Unknown	Unknowm	GSE95166	NPC	Arraystar Human LncRNA microarray V2.0 (Agilent_033010 Probe Name version)	4	4
Lyu et al. [23]	24606633	GSE43039	NPC	CCDTM-miRNA850-version 4p1.4	20	20
Bruce et al. [9]	25738365	GSE70970	NPC	nCounter^®^ Human miRNA Assay (v1.0. Nanostring)	17	246

### Identification of DEGs

To identify DEGs between normal tissue samples and NPC samples, we analyzed the microarray data by using the Limma package [24] and a multivariate linear model of the adjusted t-statistic. The cutoff criteria were as follows: |log Fold Change| (absolute value of log2 in the fold change of gene expression) ≥ 1 and adjusted *P* value ≤ 0.01.

### Enrichment analyses of NPC

Gene ontology (GO) is the main bioinformatics tool for gene annotation and analysis of the biological processes (BPs) of genes and gene products [25], which involves annotation of BPs, molecular functions (MFs), and cellular components (CCs). GO analysis of DEGs was conducted using UpSetR [26]. The kyoto encyclopedia of genes and genomes (KEGG) is a bioinformatics database resource for determining the high-level functions and uses of cells and organisms from their genomic information [27]. To investigate the functional and pathway enrichment in GO and KEGG, we used UpSetR to identify the modules involved in biological functions. Gene set enrichment analysis (GSEA) is a knowledge-based method for the translation of genome-wide expression profiles [28]. We analyzed pathways using GSEA, and identified each functional cluster using clusterProfiler [29]. The cut-off criteria were a false discovery rate < 0.1 and P value < 0.01.

### Construction of the lncRNA-miRNA-mRNA interaction network

The lncRNA-miRNA interactions were predicted using the miRcode tool (http://www.mircode.org/), which is described as “a map of putative miRNA target sites in the long non-coding transcriptome” [30]. Three convenient online databases, namely, miRDB [31] (http://www.mirdb.org/), miRTarBas [32] (http://mirtarbase.mbc.nctu.edu.tw), and TargetScan [33] (http://www.targetscan.org), were used to predict the target mRNAs of the miRNAs. Data with five or more binding sites were retained. We selected the mRNAs at the intersection of the three databases as the predictive targets of miRNAs for the construction of lncRNA–miRNA–mRNA ceRNA networks. Two separate ceRNA networks were constructed using upregulated and downregulated RNAs, and these were visualized using Cytoscape (https://cytoscape.org/, version 3.7.2), a popular online bioinformatics database [34].

### Protein-protein interaction network construction

The protein–protein interaction (PPI) network was predicted using the Search Tool for the Retrieval of Interacting Genes [35] (STRING; http://string-db.org, version 11.3) online database. Significant insights into the underlying mechanisms of NPC can be provided by investigating the interactions between proteins. All DEGs of mRNAs were predicted using STRING, and a comprehensive score over 0.4 was regarded as statistically significant. Cytoscape (version 3.7.2; https://cytoscape.org/) was used to visualize biological network and integrate the data [36]. The Molecular Complex Detection (MCODE; version 1.6) algorithm of Cytoscape was used for detecting densely connected regions in the PPI network, which represented the most closely related gene sets among the DEGs [37]. NetworkAnalyst (https://www.networkanalyst.ca/faces/home.xhtml), a visual analysis platform for the network-based meta-analysis of gene expression data [38], was used to visualize the proportion of DEGs. CytoHubba, a Cytoscape plugin [39], was used to filter out the top 20 hub genes with the strongest connections to the other genes in the merged network.

### Statistical analysis

Most statistical analyses were conducted using the bioinformatics tools mentioned above, and the version of R software is version 3.6.1. Differential expression levels of mRNA, miRNA and lncRNA were obtained using a two-tailed Student’s t-test. For the identification of DEG, Benjamini and Hochberg False Discovery Rate method were performed to adjust P-value. Functional and pathway enrichment analyses were analyzed by the hypergeometric test and Bonferroni correction. Variables were expressed as mean ± standard deviation. A P value < 0.01 was regarded as notably significant.

## Results

### Data collection and preprocessing

**Fig. 1:**
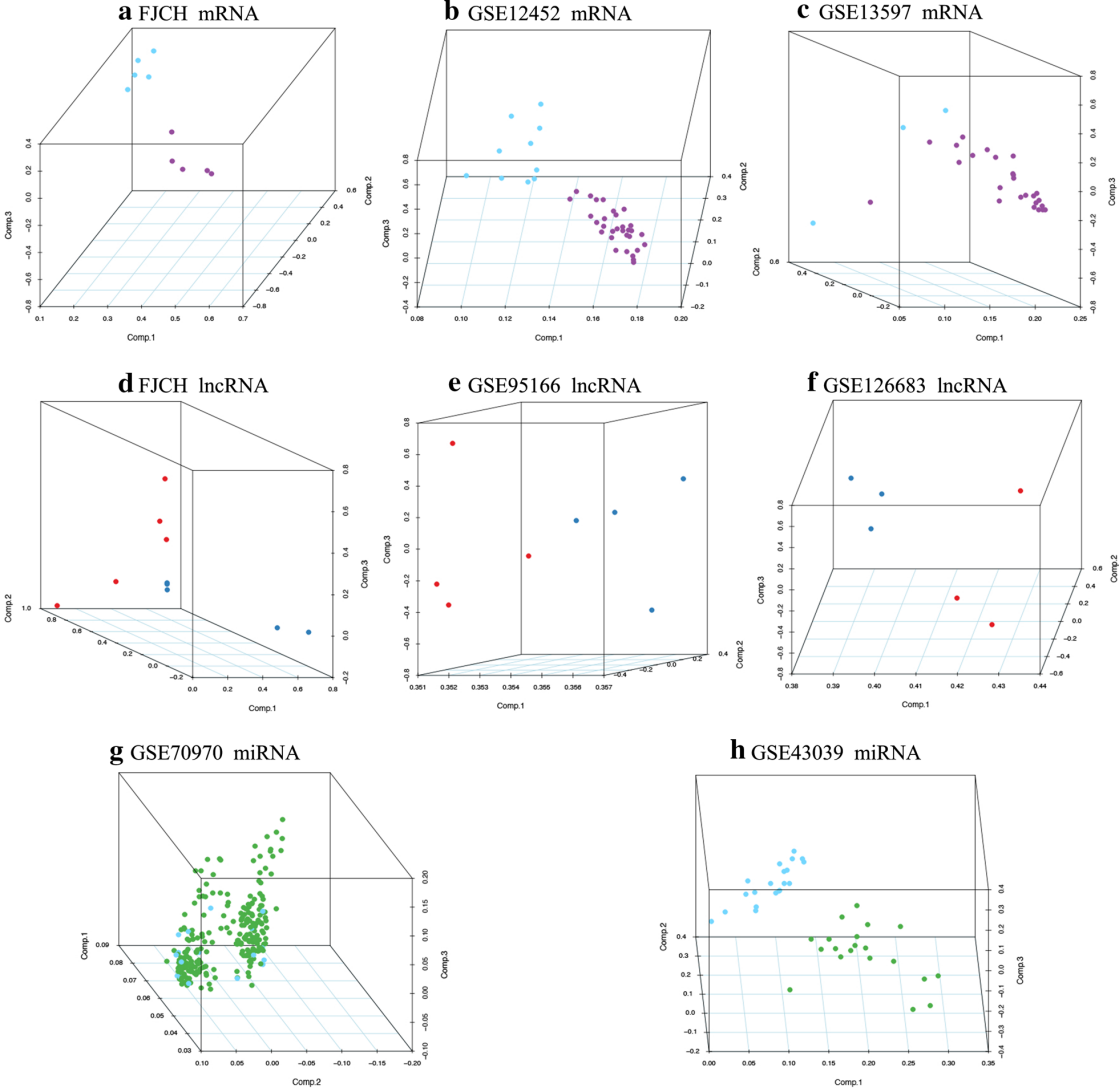
Principal component analysis (PCA) showing the clustering of mRNA, long non-coding RNA (lncRNA), and microRNA (miRNA) expression matrices in different samples and different datasets. **a**–**c** PCA of mRNA expression between the nasopharyngeal carcinoma (NPC) cluster and normal tissue cluster in the FJCH (**a**), GSE12452 (**b**), and GSE13597 (**c**) datasets. The purple dots represent the NPC samples, and the blue dots represent the normal tissue (control) samples. **d**–**f** PCA of lncRNA expression between the NPC cluster and normal cluster in the FJCH (**d**), GSE95166 (**e**), and GSE126683 (**f**) datasets. The blue dots represent the NPC samples, and the red dots represent the normal tissue (control) samples. **g**, **h** PCA of miRNA expression between the NPC cluster and normal cluster in the GSE70970 (**g**) and GSE43039 (**h**) datasets. The green dots represent the NPC samples, and the blue dots represent the normal tissue (control) samples

To determine whether there was clustering or outliers in the sample set, the differences between the clustering of the mRNA (Fig. 1a–c), lncRNA (Fig. 1d–f), and miRNA (Fig. 1g, h) expression matrixes of the NPC and normal tissue samples in different datasets were examined using three‐dimensional principal component analysis (PCA). The results showed that NPC was well distinguished from the normal tissue samples.

### Identification of DEGs in NPC

**Fig. 2:**
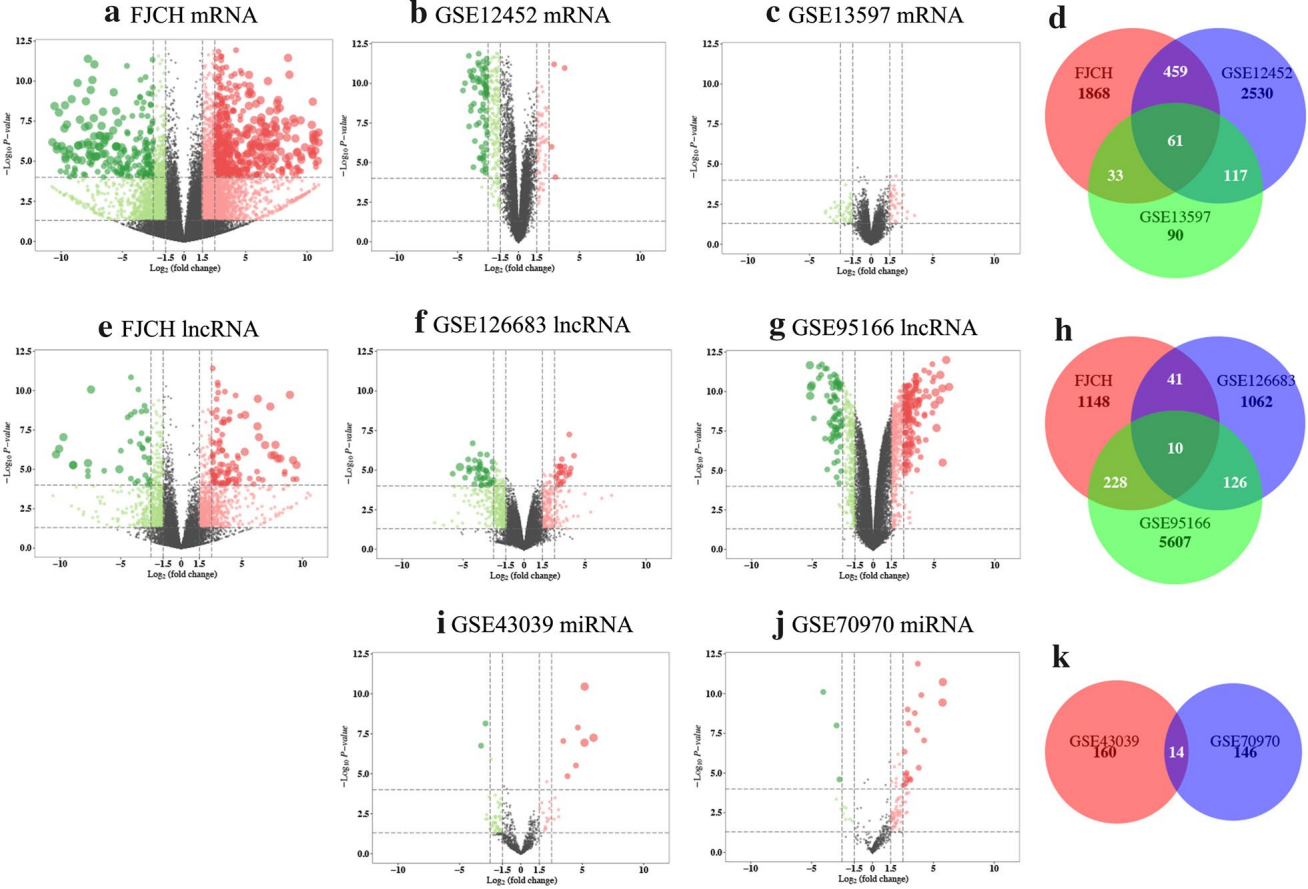
Volcano plots of the distributions of DEGs in different datasets. **a**–**c** Volcano plots of the distributions of DEmRNAs in the FJCH (**a**), GSE12452 (**b**), and GSE13597 (**c**) datasets. **e**–**g** Volcano plots of the distributions of DElncRNAs in the FJCH (**e**), GSE126683 (**f**), and GSE95166 (**g**) datasets. **i**, **j** Volcano plots of the distributions of DEmiRNAs in the GSE43039 (**i**), and GSE70970 (**j**) datasets. Differentially expressed genes (DEGs) were those with a fold change of > 2 and a P‐value of < 0.01 in the mRNA expression matrix, lncRNA expression matrix and miRNA expression matrix. Upregulated DEGs are mapped as red spots, and downregulated DEGs are mapped as green spots. Genes without notable variation are labelled as black spots. **d** Venn diagram of the DEGs among the mRNA expression datasets FJCH, GSE12452, and GSE13597. **h** Venn diagram of the DEGs among the lncRNA expression datasets FJCH, GSE43039, and GSE70970. **k** Venn diagram of the DEGs among the miRNA expression datasets GSE43039 and GSE70970

To identify the DEGs in NPC, the mRNA, lncRNA, and miRNA expression profiles were analyzed using the Limma package. The results showed that 3664 mRNAs, 4068 lncRNAs, and 265 miRNAs were dissimilarly expressed (|logFC| ≥ 1, adjusted P ≤ 0.01; Fig. 2) between the NPC and normal tissues. Of these, 2181 mRNAs, 2087 lncRNAs, and 175 miRNAs were significantly upregulated, while 1483 mRNAs, 1981 lncRNAs, and 90 miRNAs were significantly downregulated. In total, 61 DEGs were shared among the three mRNA datasets (Fig. 2d), 10 differentially expressed lncRNAs (DElncRNAs) were shared among the three lncRNA datasets (Fig. 2h), and 14 differentially expressed miRNAs (DEmiRNAs) were shared among the two miRNA datasets (Fig. 2k). Those DEGs may provide new insight into the biological mechanisms of NPC and serve as potential therapeutic targets for NPC. Functional roles of 10 DElncRNAs shared among the three lncRNA datasets are provided in Additional file 1: Table S1, and functional roles of 14 DEmiRNAs shared among the two miRNA datasets are provided in Additional file 2: Table S2.

### Construction of the ceRNA network

**Fig. 3:**
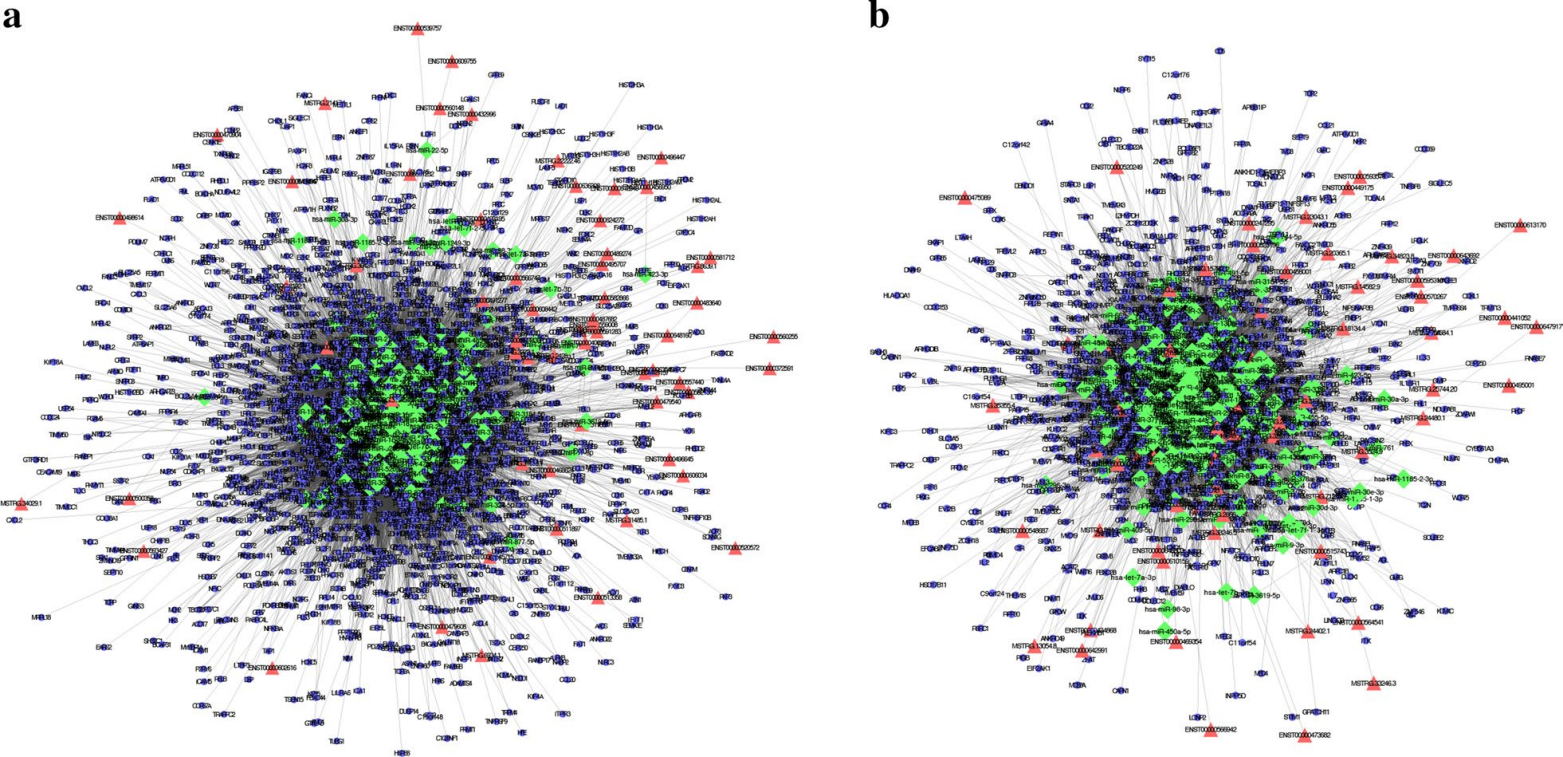
Interaction networks of mRNA-miRNA-lncRNA in nasopharyngeal carcinoma (NPC). **a** A ceRNA network of upregulated genes. **b** A ceRNA network of downregulated genes. The blue, red and green nodes represent predictive miRNAs, predictive long non-coding RNAs (lncRNAs), and predictive microRNAs (miRNAs), respectively

To explore the role of miRNAs and corresponding target mRNAs, as well as corresponding lncRNAs in NPC, we predicted the target mRNAs of the DEmiRNAs, and lncRNAs that may have interrelations with miRNAs. The results may help to better explain the critical regulatory functions of miRNAs, mRNAs, and lncRNAs. The interaction of up-regulated and down-regulated miRNAs with DElncRNAs was predicted based on miRcode. The prediction of target mRNAs of up-regulated and down-regulated miRNAs was performed using three databases, miRDB, miRTarBas, and TargetScan. LncRNAs, miRNAs and mRNAs were included in the up-regulated and down-regulated lncRNA-miRNA-mRNA ceRNA networks respectively (Fig. 3a, b). The blue, red and green nodes represent miRNAs, lncRNAs, and miRNAs, respectively. Additional files 3, 4: Tables S3, S4 shows the details of the interactions of the up-regulated and down-regulated miRNAs and mRNAs, respectively. Additional file 5, 6: Tables S5 and S6 shows the details of the interactions of the up-regulated and down-regulated miRNAs and lncRNAs, respectively.

### GO and KEGG analyses of DEGs

**Fig. 4:**
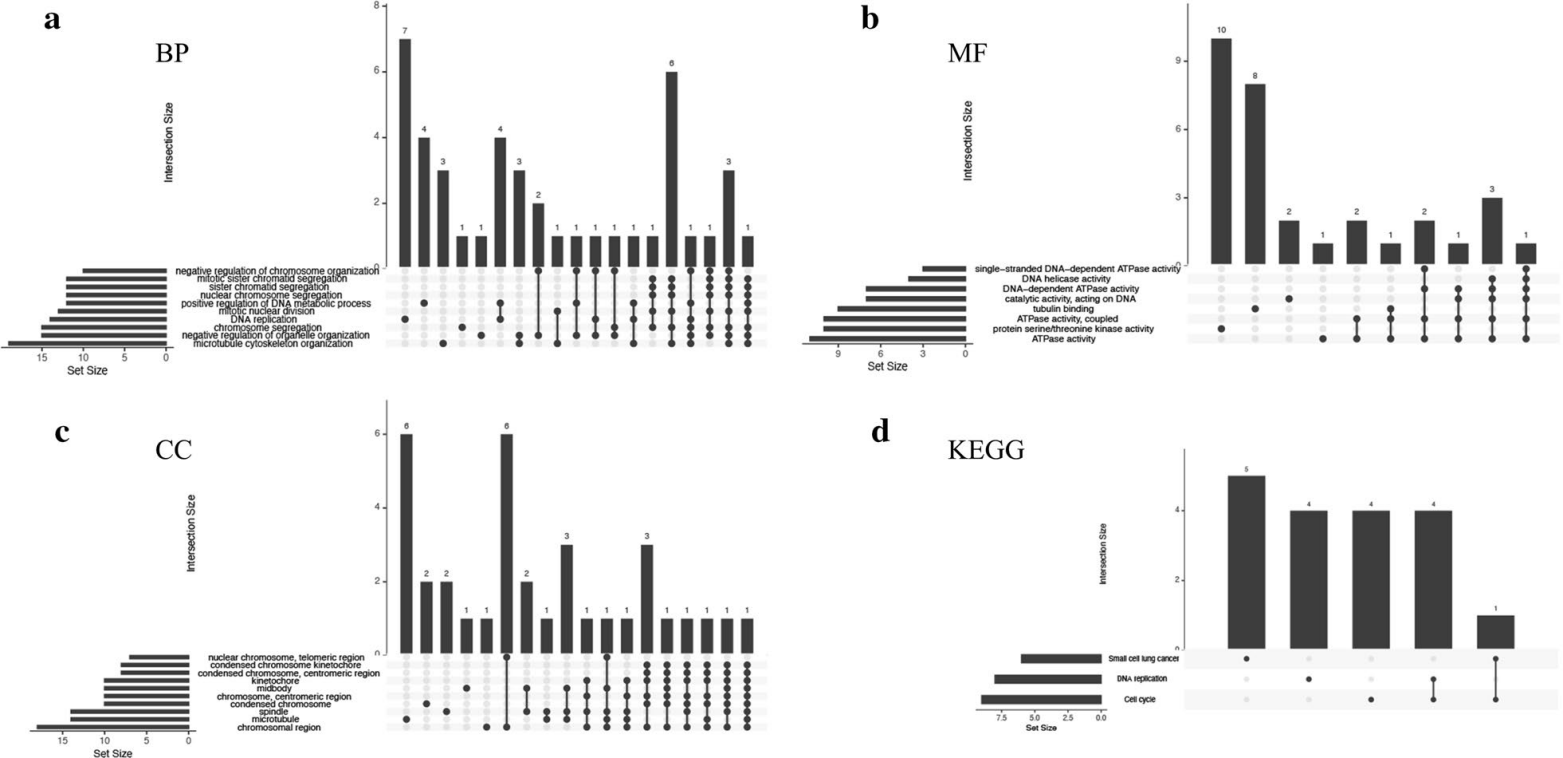
UpSetR plots showing the distributions of the gene ontology (GO) annotations associated with the differentially expressed genes (DEGs) in nasopharyngeal carcinoma (NPC). **a** Biological processes. **b** Molecular functions. **c** Cellular components. **d** UpSetR plot showing the distribution of pathways associated with the DEGs in NPC based on kyoto encyclopedia of genes and genomes (KEGG) analysis

To further analyze the possible functions of the 61 DEGs, we conducted biological analyses by using clusterProfiler and UpSetR. The results suggested that the DEGs were significantly enriched in GO and KEGG terms. The GO analysis showed that the following BPs were notably enriched among the DEGs: chromosome segregation, nuclear chromosome segregation, sister chromatid segregation, mitotic sister chromatid segregation, negative regulation of chromosome organization (Fig. 4a). The following MFs were largely enriched in ATPase activity, protein serine/threonine kinase activity, ATPase activity (coupled), tubulin binding, catalytic activity (acting on DNA), DNA dependent ATPase activity, DNA helicase activity, and single-stranded DNA dependent ATPase activity (Fig. 4b). Finally, the following CCs were found to be largely enriched in the chromosomal region, condensed chromosome, chromosome (centromeric region), condensed chromosome (centromeric region), nuclear chromosome (telomeric region), and condensed chromosome kinetochore (Fig. 4c). The KEGG pathway analysis suggested that DEGs in NPC were largely enriched in the cell cycle, DNA replication, and small cell lung cancer (Fig. 4d). The results suggested that chromosomal dysfunction was closely related to the development of NPC.

### GSEA of NPC-related genes

**Fig. 5:**
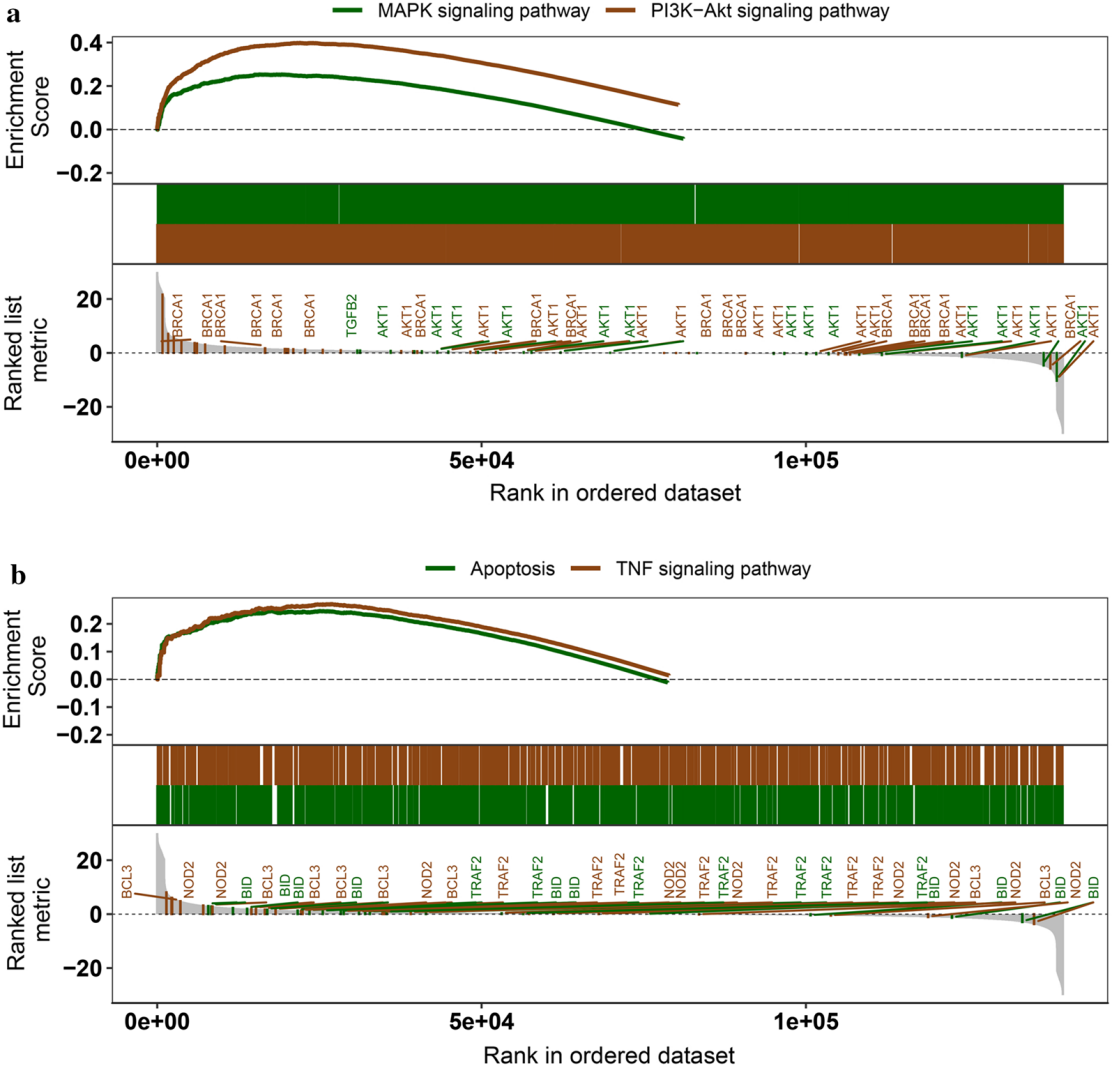
Gene set enrichment analysis (GSEA) of the gene expression profiles of the FJCH dataset. **a** GSEA shows that the mitogen-activated protein kinase (MAPK) pathway and the phosphatidylinositol 3-kinase/protein kinase B (PI3K-AKT) pathway are concentrated in nasopharyngeal carcinoma (NPC). **b** GSEA reveals that the apoptosis pathway and the tumor necrosis factor (TNF) pathway are concentrated in NPC

To explore the biological functions of the DEGs involved in NPC, GSEA was applied. The mRNA expression profile of the FJCH dataset was subjected to GSEA by means of clusterProfiler. The analysis showed that the following biological pathways were over-represented in the NPC tissues as compared to the normal tissues : the mitogen-activated protein kinase (MAPK) signaling pathway, the phosphatidylinositol-3 OH kinase/protein kinase B (PI3K-Akt) signaling pathway (Fig. 5a), the apoptotic pathway, and the tumor necrosis factor (TNF) signaling pathway (Fig. 5b). The pathways found our study were involved with cancer progression, metastasis, and apoptosis.

### PPI network analysis of DEGs

**Fig. 6:**
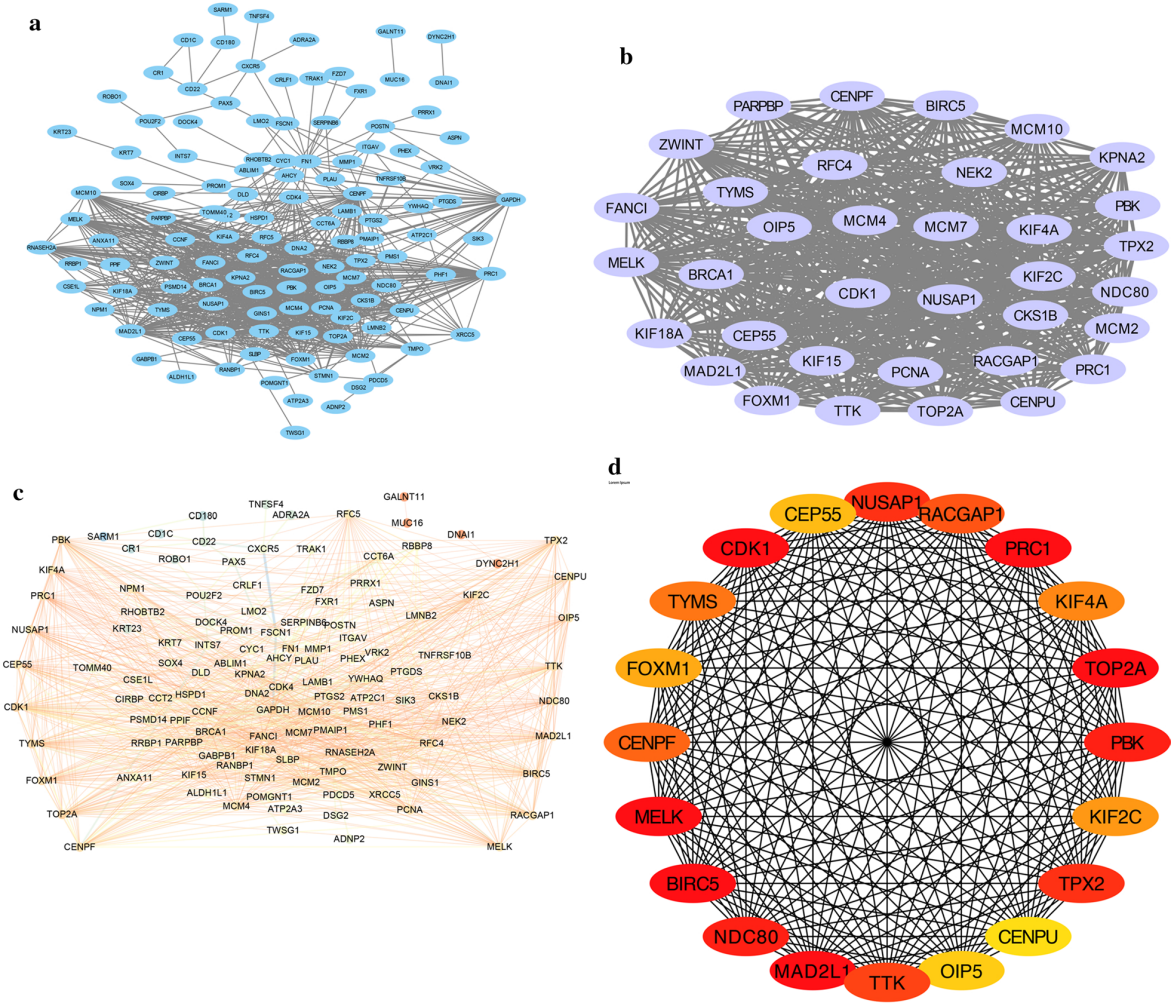
Protein-protein interaction (PPI) networks. **a** A PPI network of differentially expressed genes (DEGs) constructed using STRING software. **b** Most relevant gene sets in the PPI network extracted using MCODE. **c** Further analysis of DEGs using NetworkAnalyzer. **d** The top 20 hub genes with the most correlations identified using CytoHubba

The STRING database was used (version: 11.3) to explore the PPI network based on the correlations among the 61 DEGs in NPC. The obtained data were then examined using Cytoscape software. The PPI network of DEGs was constructed using MCODE to obtain the vital gene module. The NetworkAnalyzer plugin was applied to further analyze the PPI network according to the scores. The cytoHubba plugin was used to analyze the hub genes associated with NPC, and the following genes with the top 20 grades were deemed to be hub genes: NUSAP1, RACGAP1, PRC1, KIF4A, TOP2A, PBK, KIF2C, TPX2, CENPU, OIP5, TTK, MAD2L1, NDC80, BIRC5, MELK, CENPF, FOXM1, TYMS, CDK1, and CEP55 (Fig. 6). Those genes may contribute to the investigation of biological mechanisms and uncover underlying therapeutic targets for NPC.

### GO and KEGG analyses of hub genes

**Fig. 7:**
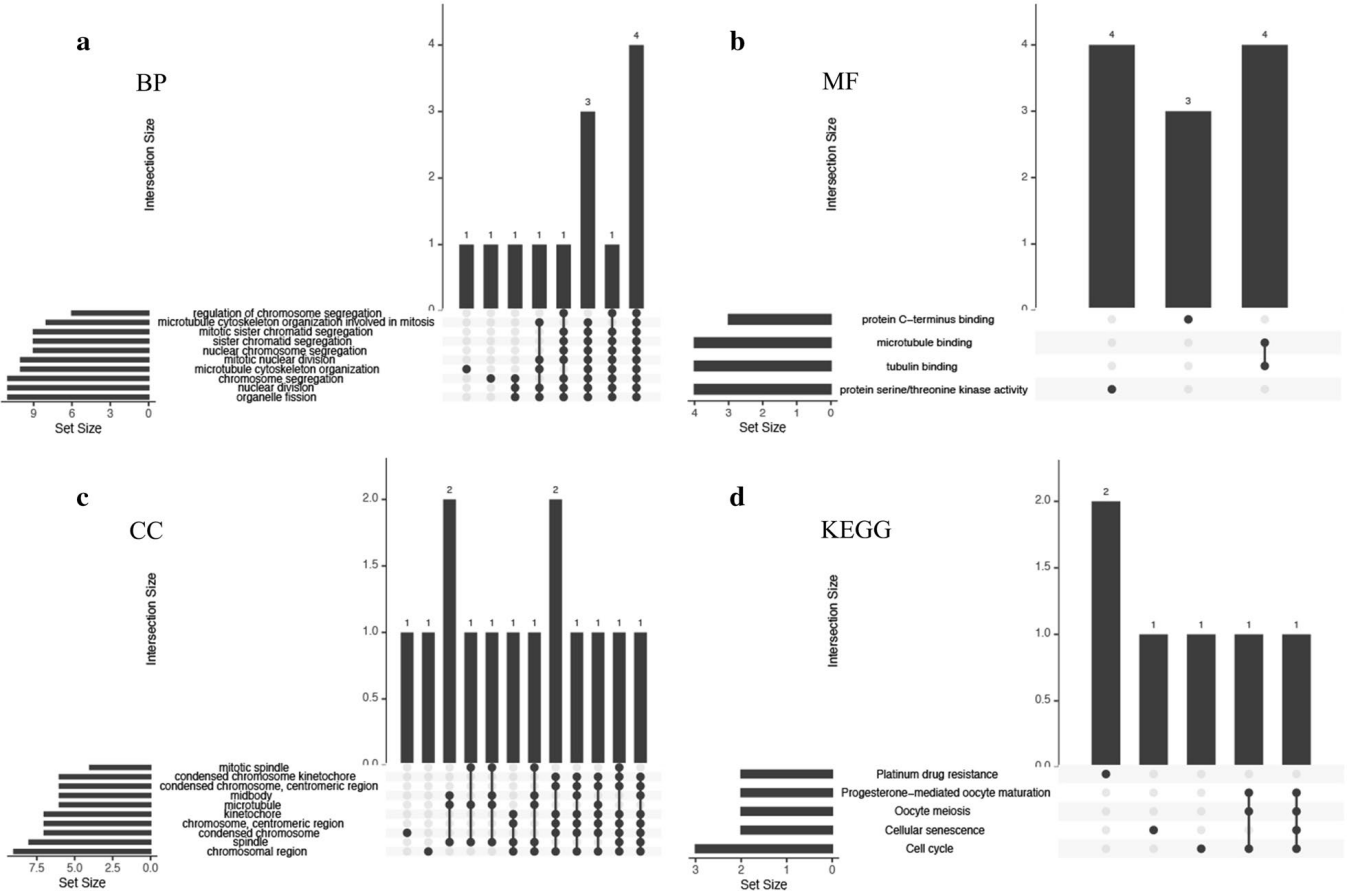
UpSetR plots showing the distributions of the gene ontology (GO) annotations associated with the hub genes of nasopharyngeal carcinoma (NPC) in the case of **a** biological processes, **b** molecular functions, and **c** cellular components and **d** UpSetR plot showing the distribution of pathways associated with the hub genes of NPC, based on kyoto encyclopedia of genes and genomes (KEGG) analysis

To analyze the functions of the top 20 hub genes, we again conducted biological analyses by using clusterProfiler and UpSetR. The results suggested that the hub genes were significantly enriched in GO and KEGG terms. GO analysis showed that changes in the following BPs of hub genes were notably enriched in chromosome segregation, nuclear chromosome segregation, sister chromatid segregation, mitotic sister chromatid segregation, microtubule cytoskeleton organization involved in mitosis, and regulation of chromosome segregation (Fig. 7a). In addition, the changes in the following MFs were mainly enriched in protein serine/threonine kinase activity, tubulin binding, microtubule binding, and protein C-terminus binding (Fig. 7b). Finally, changes in the following CCs of DEGs were enriched in the chromosomal region, spindle, condensed chromosome, chromosome (centromeric region), kinetochore, microtubule, midbody, condensed chromosome (centromeric region), condensed chromosome kinetochore, and mitotic spindle (Fig. 7c). KEGG pathway analysis indicated that the DEGs in NPC were mainly enriched in the cell cycle, cellular senescence, oocyte meiosis, progesterone-mediated oocyte maturation, and platinum drug resistance (Fig. 7d). Enrichment analyses of the hub genes were similar to the results of the analyses of the DEGs. Hence, the findings obviously suggested that chromosomal dysfunction was a vital contributor to the tumorigenesis of NPC.

## Discussion

In this work, we performed a comprehensive analysis of the full transcriptome sequencing dataset of Fujian Cancer Hospital and six microarray datasets downloaded from the GEO repository to uncover DEGs between NPC tissues and normal nasopharyngeal tissues. We identified differentially expressed mRNAs (DEmRNAs), DEmiRNAs, and DElncRNAs among the seven datasets, and constructed a lncRNA-miRNA-mRNA network of NPC. GO enrichment analysis, KEGG enrichment analysis, and GSEA proved that the enriched components and pathways among the DEGs associated with NPC were inseparable from the chromosome dysfunction, MAPK signaling pathway, and PI3K-Akt signaling pathway discovered in NPC. We also identified the top 20 hub genes in the PPI network related to NPC, and the results of the enrichment analysis of the hub genes were similar to those of the DEGs.

Studies have shown that lncRNA-miRNA-mRNA networks play significant roles in the development and progression of tumors [40]. By constructing visual networks, we can see the interaction between DEGs of different molecular types. The lncRNA-miRNA-mRNA network constructed in our study indicated that in NPC, 2654 mRNAs could be regulated by 132 lncRNAs via 565 corresponding miRNAs. Li et al. identified 37 miRNAs, including 19 highly expressed miRNAs and 18 lowly expressed miRNAs, from the serum of 12 NPC patients with different radiosensitivity; these miRNAs were found to have remarkable differences between the patients (fold change ≥ 2 or ≤ 0.5 and P < 0.05) [41]. The highly expressed miRNA hsa-miR-6088 and the lowly expressed miRNA hsa-let-7f-1-3p from the above study were also found in our ceRNA networks. We also identified hsa-miR-29a-3p and hsa-miR-103a-3p as DEmiRNAs, which were recently found to act as circulating biomarkers of NPC, with fairly good diagnostic accuracy for detecting NPC as compared with controls (area under the curve > 0.7) [42]. The radioresistant NPC CNE2-IR cell line has been shown to overexpress JUN; Guo et al. identified 35 JUN-related miRNAs by using mirDIP software, including hsa-miR-200b-3p, hsa-miR-139-5p, hsa-miR-200c-3p, hsa-miR-9-5p, and hsa-miR-92b-3p [43]. Thus, JUN could promote tumorigenesis and tumor development. Qing et al. found that inhibiting c-JUN expression could enhance radiosensitivity, and induce cell cycle arrest and apoptosis [44]. The above results show that ceRNA networks can offer insights into the complex regulation patterns of NPC and potentially facilitate the individualized treatment of NPC.

GO analyses of the BPs of the DEGs associated with NPC showed that the negative regulation of chromosome segregation, nuclear chromosome segregation, sister chromatid segregation, mitotic sister chromatid segregation, and negative regulation of chromosome organization were closely associated with the oncogenesis of NPC. Among the CC annotations, chromosomal region, condensed chromosome, chromosome (centromeric region), condensed chromosome (centromeric region), condensed chromosome kinetochore, and nuclear chromosome (telomeric region) were notably related to NPC. Several studies have reported on the chromosomal aberrations involved in the carcinogenesis of NPC, including chromosomal gains or losses [45, 46], loss of heterozygosity [47], chromosomal rearrangements [48], and chromosomal imbalances [49]. In one study, loss of heterozygosity on 3p was observed in 95%–100% of primary NPC specimens and almost 75% of pre-cancerous lesions [47]. Tan et al. hypothesized that apoptosis induced by oxidative stress may lead to CAD-mediated chromosomal breakage. After incorrect DNA repair, cells that survive apoptosis may carry chromosomal rearrangements, leading to the tumorigenesis of NPC [48]. To investigate common genetic variations in NPC, Natasya et al. screened out 7 cases of NPC in the Malaysian population by the comparative genomic hybridization (CGH) technology. And the results showed chromosomal changes in all 7 NPC cases [46]. Enrichment analyses of the 20 hub genes identified in our study were greatly compliant with the results of the enrichment analyses of the DEGs. Thus, the above findings clearly implicate chromosomal dysfunction as an important contributor to the carcinogenesis of NPC.

GSEA showed that the MAPK signaling pathway, PI3K-Akt signaling pathway, apoptotic pathway, and TNF signaling pathway were the top four pathways associated with NPC. The enriched pathways identified in our investigation are related to tumor progression, metastasis, and apoptosis. The MAPK/extracellular signal-regulated kinase (ERK) pathway has been reported to be closely related to cell proliferation, differentiation, migration, senescence, and apoptosis [50]. In prostate carcinoma, intracellular chloride channels have been proved to influence cell multiplication and migration via the MAPK/ERK pathway [51]. In hepatocellular cancer, lysyl oxidase propeptide could induce apoptosis via downregulation of the MAPK/ERK pathway [52]. Recent studies have found that knockdown of amyloid β precursor protein is closely associated with the downregulation of the MAPK pathway, and this could greatly impede the neoplasia of NPC [53]. The PI3K/Akt pathway is vital to NPC progression, metastasis, and invasion [54]. After the EBV genome is introduced into the NPC cells, this pathway could be activated [55]. A study showed that inhibiting the PI3K/Akt pathway could suppress NPC cell metastasis by inducing mesenchymal-epithelial reverting transition [56]. Studies have shown that asiatic acid notably reduces the viability of cisplatin-resistant NPC cells by inducing apoptosis via the internal and external apoptotic pathways [57]. The proto-oncogene JUN is associated with the cis-regulatory lncRNA RP4-794H19.1 and is very commonly found in cancers; JUN has been linked to the TNF signaling pathway, and may be a vital gene in NPC [58]. TNF-α may be a tumor-promoting factor in NPC, as TNF-α expression has been observed in both primary NPC specimens and serum derived from NPC patients, and can significantly predict the risk of distant metastasis in NPC patients [59].

In the PPI network constructed in our study, the following 20 DEGs with many interactions were selected as vital hub genes. More observations of these genes may lead to further insights into the carcinogenesis of NPC. For example, the study of Wang et al. has revealed that lncRNA HCP5 may promote the malignant behavior of ovarian cancer cells by miR-525-5p/PRC1 crosstalk and Wnt/β-catenin pathway [60]. PRC1 has been associated with many malignant carcinomas, such as NPC [61], prostate cancer [62], and lung cancer [63]. In vitro experiments have shown that PRC1 depletion inhibits the multiplication and invasiveness of NPC, while in vivo studies have found that PRC1 inhibits the neoplasia and radioresistance of NPC [61]. Increased PBK expression in NPC patients has been positively correlated with clinical severity, specifically advanced T stage, and disease progression. Indeed, PBK overexpression is an independent prognostic factor that shortens overall survival; the malignant phenotype of NPC requires PBK, as downregulation of PBK expression can inhibit NPC cell proliferation [64]. One study found that the overexpression of miR‐372 via the downregulation of PBK not only enhances the radiosensitivity of NPC but also reduces its aggressiveness and inhibits metastasis [65]. TOP2A is closely related to cell division by selective cleavage, rearrangement, and reconnection of DNA strands. This gene is highly expressed in NPC subpopulations. Its enhanced immune expression is markedly related to advanced cancer and the invasiveness of NPC [66].

Our study has certain limitations. First, we did not perform any further verification using molecular experiments, such as western blot analysis, quantitative real‐time PCR, and immunohistochemistry, to fully clarify the roles of the predicted hub genes and signaling pathways and uncover the potential mechanisms underlying NPC. It is also necessary to conduct loss‐of‐function and gain‐of‐function studies with tissue‐type specificity and cell‐type specificity. Second, we lack corresponding clinical relevance studies and analyses based on clinical information. Third, we used seven datasets to reduce the high false-positive rate related to single microarray analysis. However, the use of many datasets may lead to inter-batch differences that cannot be avoided or removed during the analyses. Finally, it is not clear whether the prediction of biomarkers using the background-corrected matrix file was reliable, as each GEO dataset was obtained using a different correction method [67].

In summary, we performed integrated bioinformatics analyses on seven datasets to identify DEGs involved in the pathogenesis of NPC. Altogether, we uncovered 3664 DEmRNAs, 4068 DElncRNAs, 265 DEmiRNAs, and 20 hub genes that may serve as biomarkers for the diagnosis, prognosis, and therapy of NPC. GO analysis, KEGG analysis and GSEA indicated that chromosome dysfunction could underlie the pathogenesis of NPC. We also constructed a lncRNA–miRNA–mRNA network to better understand the potential biological mechanisms among the identified genes. Our results may provide new targets for understanding the molecular mechanisms of NPC.

## Conclusions

In our present study, we found several DEGs and their biological processes, signalling pathways, and predicted the target mRNAs of the DEmiRNAs, as well as lncRNAs that may have interrelations with DEmiRNAs by performing integrated bioinformatics analyses. Our study uncovered 20 hub genes via the PPI network, which may contribute to the exploration of biological mechanisms underlying NPC and identify potential therapeutic targets for NPC. Nevertheless, specific pathogenesis and molecular targets still need to be further verified through molecular experiments.

## Supplementary information

**Additional file 1: Table S1.** Functional roles of 10 differentially expressed lncRNAs shared among the three lncRNA datasets.

**Additional file 2: Table S2.** Functional roles of 14 differentially expressed miRNAs shared among the two miRNA datasets.

**Additional file 3: Table S3.** Details of the interactions of the up-regulated miRNAs and mRNAs.

**Additional file 4: Table S4.** Details of the interactions of the down-regulated miRNAs and mRNAs.

**Additional file 5: Table S5.** Details of the interactions of the up-regulated miRNAs and lncRNAs

**Additional file 6: Table S6.** Details of the interactions of the down-regulated miRNAs and lncRNAs.

## Data Availability

The datasets generated and/or analyzed during the current study are available in the [GEO] repository, [http://www.ncbi.nlm.nih.gov/geo] [68]. Reference Other datasets used and/or analyzed during the current study are available from the corresponding author on reasonable request.

## Abbreviations

NPC: Nasopharyngeal carcinoma
lncRNAs: Long non-coding RNAs
miRNAs: MicroRNAs
GO: Gene ontology
KEGG: Kyoto encyclopedia of genes and genomes
GSEA: Gene set enrichment analysis
ceRNA: Competing endogenous RNA
PPI: Protein–protein interaction
DEGs: Differentially expressed genes
STRING: Search Tool for the Retrieval of Interacting Genes
MCODE: Molecular Complex Detection
DElncRNAs: Differentially expressed lncRNAs
DEmiRNAs: Differentially expressed miRNAs
MAPK: Mitogen-activated protein kinase
PI3K-Akt: Phosphatidylinositol-3 OH kinase/protein kinase B
TNF: Tumor necrosis factor
EBV: Epstein–Barr virus
GEO: Gene Expression Omnibus
BPs: Biological processes
MFs: Molecular functions
CCs: Cellular components
PCA: Three‐dimensional principal component analysis
ERK: Extracellular signal-regulated kinase
DEmRNAs: Differentially expressed mRNAs
