# Supplementary material for: Comprehensive analysis of key genes associated with ceRNA networks in nasopharyngeal carcinoma based on bioinformatics analysis
Source: Cancer Cell Int. 2020 Aug 26;20:408. doi: 10.1186/s12935-020-01507-1 (PMC7448472; doi:10.1186/s12935-020-01507-1)
Supplement: Supplementary file 1 — Additional file 1: Table S1. Functional roles of 10 differentially expressed lncRNAs shared among the three lncRNA datasets. [file 12935_2020_1507_MOESM1_ESM.docx]

**TableS1: Functional roles of 10 differentially expressed lncRNAs shared among the three lncRNA datasets.**

| **NO.** | **Gene symbol** | **Function** |
| --- | --- | --- |
| 1 | LINC02195 | Plays a key role in regulating the expression of MHC I molecules, and it was an immune-related lncRNA and positively involved in an increased T cell density^1^ |
| 2 | SET | NA |
| 3 | UCK2 | In the cells of colorectal, UCK2 inhibition directly resulted in reduced 18S RNA biosynthesis and concomitant cell cycle arrest^2^ |
| 4 | DNAH6 | Required for motile cilia function mediating airway clearance and left/right patterning^3^ |
| 5 | AC005332.5 | NA |
| 6 | TMPO | Promotes the proliferation of hepatocellular cancer cells, migration and invasion by sponging miR-329-3p to stimulate FOXK1-mediated AKT/mTOR pathway^4^ |
| 7 | AC008124.1 | NA |
| 8 | FUBP1 | Repressed p21 mRNA stabilization and regulated pro-apoptotic genes transcription, acted as an anti-apoptotic factor in hepatocellular carcinoma^5^ |
| 9 | ENO1 | Correlated with the regulation of cancer cell proliferation and metastasis^6^ |
| 10 | LINC01089 | Predicts clinical prognosis and inhibits cell proliferation and invasion through the Wnt/β-catenin signaling pathway in breast cancer^7^ |

**References:**

1. Li H, Xiong HG, Xiao Y, et al. Long Non-coding RNA LINC02195 as a Regulator of MHC I Molecules and Favorable Prognostic Marker for Head and Neck Squamous Cell Carcinoma. Frontiers in Oncology. 2020;10.

doi: 10.3389/fonc.2020.00615.

1. Yu S, Li X, Guo X, et al. UCK2 upregulation might serve as an indicator of unfavorable prognosis of hepatocellular carcinoma. IUBMB Life. 2019 01;71(1). doi: 10.1002/iub.1941.
2. Li Y, Yagi H, Onuoha EO, et al. DNAH6 and Its Interactions with PCD Genes in Heterotaxy and Primary Ciliary Dyskinesia. PLoS Genet. 2016 Feb;12(2). doi: 10.1371/journal.pgen.1005821.
3. Guo X, Wang Y. LncRNA TMPO-AS1 promotes hepatocellular carcinoma cell proliferation, migration and invasion through sponging miR-329-3p to stimulate FOXK1-mediated AKT/mTOR signaling pathway. Cancer Medicine. 2020 May 27. doi: 10.1002/cam4.3046.
4. Han T, Wu Y, Hu X, et al. NORAD orchestrates endometrial cancer progression by sequestering FUBP1 nuclear localization to promote cell apoptosis. Cell Death & Disease. 2020 Jun 18;11(6). doi: 10.1038/s41419-020-2674-y.
5. Zhang J, Li H, Miao L, et al. Silencing of ENO1 inhibits the proliferation, migration and invasion of human breast cancer cells. Journal of BUON. 2020 Mar-Apr;25(2).
6. Yuan H, Qin Y, Zeng B, et al. Long noncoding RNA LINC01089 predicts clinical prognosis and inhibits cell proliferation and invasion through the Wnt/β-catenin signaling pathway in breast cancer. OncoTargets and Therapy. 2019;12. doi: 10.2147/OTT.S208830.
