# Supplementary material for: Comprehensive analysis of key genes associated with ceRNA networks in nasopharyngeal carcinoma based on bioinformatics analysis
Source: Cancer Cell Int. 2020 Aug 26;20:408. doi: 10.1186/s12935-020-01507-1 (PMC7448472; doi:10.1186/s12935-020-01507-1)
Supplement: Supplementary file 2 — Additional file 2: Table S2. Functional roles of 14 differentially expressed miRNAs shared among the two miRNA datasets. [file 12935_2020_1507_MOESM2_ESM.docx]

**TableS2: Functional roles of 14 differentially expressed miRNAs shared among the two miRNA datasets.**

| **NO.** | **Gene symbol** | **Function** |
| --- | --- | --- |
| 1 | hsa-miR-205 | Inhibits cell proliferation and anchorage independent growth and cell invasion^1^ |
| 2 | hsa-miR-184 | An oncogenic miRNA with an antiapoptotic effect^2^ |
| 3 | hsa-miR-452 | Correlated with tumor progression, underlying tumor suppressive gene^2^ |
| 4 | hsa-miR-211 | Triggers an autophagy-dependent apoptosis in cervical cancer cells^3^ |
| 5 | hsa-miR-29b | Over-expressed in renal cancer cell line 786-O expressing exogenous VHL, which could then target the TIS11B transcript to repress its expression under normoxia^4^ |
| 6 | hsa-miR-203 | The targets genes regulated by hsa-miR-203 were related to cell cycle function and pathway, especially in the phase of G1/S^5^ |
| 7 | hsa-miR-16 | Controls myoblast proliferation and apoptosis via directly suppressing Bcl2 and FOXO1 activities^6^ |
| 8 | hsa-miR-140 | Affects the migration and invasion of hypopharyngeal carcinoma cells by down-regulating ADAM10 expression^7^ |
| 9 | hsa-mir-181d | Up-regulated to control expression a tumor suppressor gene, PTEN to protect the cancer cell from apoptosis^8^ |
| 10 | hsa-miR-143 | An oncosuppressor many cancer^9^ |
| 11 | hsa-miR-34c | The downregulation of miR-34c expression was involved in a small increase in cellular proliferation and a significant increase in cell migration^10^ |
| 12 | hsa-miR-101 | Hypoxia-induced hsa-miR-101 promotes glycolysis by targeting TIGAR mRNA in clear cell renal cell carcinoma^11^ |
| 13 | hsa-miR-625 | Serve as prognostic and predictive markers for survival of bile duct cancer patients and could potentially be provided as targets for future therapy^12^. |
| 14 | has-miR-141 | Closely involved in the carcinogenesis of COAD^13^ |

**References:**

1. Manzanarez-Ozuna, Dora-Luz Flores, Everardo Gutiérrez-López, et al. Model based on GA and DNN for prediction of mRNA-Smad7 expression regulated by miRNAs in breast cancer. Theoretical Biology and Medical Modelling. 2018 12 29;15(1). doi: 10.1186/s12976-018-0095-8
2. Xiao B, Zhang W, Chen L, et al. Analysis of the miRNA–mRNA–lncRNA network in human estrogen receptor-positive and estrogen receptor-negative breast cancer based on TCGA data. Gene. 2018 Jun 05;658.

doi: 10.1016/j.gene.2018.03.011

1. Liu S, Wang H, Mu J, et al. MiRNA-211 triggers an autophagy-dependent apoptosis in cervical cancer cells: regulation of Bcl-2. Naunyn-Schmiedeberg's Archives of Pharmacology. 2020 03;393(3).

doi: 10.1007/s00210-019-01720-4.

1. Sinha S, Dutta S, Datta K, et al. Von Hippel-Lindau gene product modulates TIS11B expression in renal cell carcinoma: impact on vascular endothelial growth factor expression in hypoxia. The Journal of Biological Chemistry. 2009 Nov 20;284(47). doi: 10.1074/jbc.M109.058065.
2. Wang HB, Jiang ZB, Li M. Research on the typical miRNA and target genes in squamous cell carcinoma and adenocarcinoma of esophagus cancer with DNA microarray. Pathology & Oncology Research. 2014 Apr;20(2).

doi: 10.1007/s12253-013-9688-z.

1. Bladen JC, Wang J, Sangaralingam A, et al. MicroRNA and transcriptome analysis in periocular Sebaceous Gland Carcinoma. Scientific Reports. 2018 05 14;8(1). doi: 10.1038/s41598-018-25900-z.
2. Jing P, Sa N, Xu W. MiR-140-5p affects the migration and invasion of Hypopharyngeal Carcinoma cells by downregulating ADAM10 expression. Chinese Journal of Otorhinolaryngology Head and Neck Surgery. 2016 Mar;51(3). doi: 10.3760/cma.j.issn.1673-0860.2016.03.007.
3. Ma C, Miao C, Wang C, et al. Role of MicroRNAs in carcinogenesis that potential for biomarker of endometrial cancer. Cancer Biomark. 2019;26(1).

doi: 10.3233/CBM-182279.

1. Boubaker NS, Spagnuolo M, Trabelsi N, et al. MiR-143 expression profiles in urinary bladder cancer: correlation with clinical and epidemiological parameters. Molecular Biology Reports. 2020 Feb;47(2).

doi: 10.1007/s11033-019-05228-1.

1. Sommerová L, Fraňková H, Anton M, et al. Expression and Functional Characterization of miR-34c in Cervical Cancer. Klin Onkol. 2018;31.

doi: 10.14735/amko20182S82

1. Xu X, Liu C, Bao J. Hypoxia-induced hsa-miR-101 promotes glycolysis by targeting TIGAR mRNA in clear cell renal cell carcinoma. Molecular Medicine Reports. 2017 Mar;15(3). doi: 10.3892/mmr.2017.6139.
2. Wang M, Wen TF, He LH, et al. A six-microRNA set as prognostic indicators for bile duct cancer. International Journal of Clinical and Experimental Medicine. 2015;8(10). doi: 10.00000/26770318.
3. Wang JY, Wang CL, Wang XM, et al. Comprehensive analysis of microRNA/mRNA signature in colon adenocarcinoma. European Review for Medical and Pharmacological Sciences. 2017 05;21(9). doi:10.00000/28537673.
